# Type 2 diabetes progression in an adult Ugandan population with new-onset diabetes: an observational prospective study

**DOI:** 10.1186/s12875-023-02169-4

**Published:** 2023-10-19

**Authors:** Davis Kibirige, Isaac Sekitoleko, William Lumu, Moffat J. Nyirenda

**Affiliations:** 1 Medical Research Council/Uganda Virus Research Institute and London School of Hygiene and Tropical Medicine Uganda Research Unit, Non-Communicable Diseases Program, Entebbe, Uganda; 2 Department of Non-Communicable Diseases Epidemiology, Faculty of Epidemiology and Population Health, London School of Hygiene and Tropical Medicine, London, UK; 3 Department of Medicine, Uganda Martyrs Hospital Lubaga, Kampala, Uganda; 4 Department of Medicine, Mengo Hospital, Kampala, Uganda

**Keywords:** Adult patients, New-onset type 2 diabetes, Short-term diabetes progression, Sub-Saharan Africa, Uganda

## Abstract

**Background:**

The rate of progression of type 2 diabetes following diagnosis varies across individuals and populations. Studies investigating the progression of type 2 diabetes in adult African populations with newly diagnosed diabetes are limited. We aimed to investigate the prevalence and predictors of short-term (one year) diabetes progression in an adult Ugandan population with new-onset type 2 diabetes (type 2 diabetes diagnosed in < 3 months) initiated on oral hypoglycaemic agents (OHA).

**Methods:**

Two hundred and seven adult participants with type 2 diabetes diagnosed within the previous three months were followed up for 12 months. We investigated the association of specific demographic, clinical, and metabolic characteristics, and short-term diabetes progression (defined as glycated haemoglobin or HbA1c ≥ 8% on ≥ 2 OHA and/or treatment intensification).

**Results:**

One hundred sixteen participants (56%) completed the follow-up period. Sixty-four participants (55.2%, 95% CI 45.7–64.4) showed evidence of diabetes progression during the 12-month period of follow-up. An HbA1c ≥ 8% on ≥ 2 OHA and treatment intensification were noted in 44.8% and 29.3% of the participants, respectively. On multivariate analysis, only the female gender (AOR 3.2, 95% CI 1.1–9.2, *p* = 0.03) was noted to be independently associated with short-term diabetes progression.

**Conclusion:**

Short-term diabetes progression was relatively common in this study population and was independently associated with the female gender. Early intensified diabetes therapy in adult Ugandan female patients with new-onset type 2 diabetes should be emphasised to avert rapid short-term diabetes progression.

**Supplementary Information:**

The online version contains supplementary material available at 10.1186/s12875-023-02169-4.

## Introduction

Type 2 diabetes is a heterogeneous and progressive metabolic condition, whose clinical course after diagnosis varies across individuals and populations [1]. The hallmark of type 2 diabetes progression is the progressive decline of pancreatic beta-cell mass and secretory function with subsequent worsening glycaemia, secondary oral hypoglycaemic agent failure, and initiation of insulin therapy [2, 3].

Several prospective and retrospective studies that have investigated diabetes progression in Caucasian and Asian populations with type 2 diabetes have reported young age at diagnosis, high body mass index or BMI (which is weight in kg divided by height in meters squared), low pancreatic beta-cell function, markers of metabolic syndrome (high serum triglycerides or TGL and low high-density lipoprotein cholesterol or HDLC), high baseline glycated haemoglobin (HbA1c), islet autoantibody positivity, and specific genetic variants linked to insulin resistance as predictors of short- and long-term diabetes progression [4–8].

While considerable information on the predictors of progression of type 2 diabetes is available in Caucasian and Asian populations, similar studies in black African adult populations with new-onset type 2 diabetes are limited. Understanding the phenotypic characteristics that predict rapid diabetes progression in adult patients with type 2 diabetes in Uganda will be important in guiding optimal intensified individualised diabetes therapy in adult Ugandans with newly diagnosed type 2 diabetes.

In this observational prospective study, we followed up a cohort of adult patients with newly diagnosed type 2 diabetes that were enrolled in the Uganda Diabetes Phenotype (UDIP) study for a total of 12 months to determine diabetes progression and its associated predictors. Because of the predominance of pancreatic beta-cell dysfunction in adult Ugandan patients with type 2 diabetes [9], we hypothesised that markers of pancreatic beta-cell dysfunction (fasting C-peptide and Homeostatic Model assessment 2 of beta-cell function or HOMA2-%B) and low BMI will be associated with short-term diabetes progression in this study population.

## Methods

### Study design, setting, and participants

This observational prospective study was conducted in seven tertiary public and private not-for-profit mission or church-founded hospitals in Central and Southwestern Uganda between October 2019 and August 2021. These hospitals were selected because they serve urban, peri-urban, and rural populations, and have functional outpatient adult diabetes clinics.

A total of 207 adult patients with newly diagnosed type 2 diabetes initiated on oral hypoglycaemic agents (OHA) and enrolled in the main UDIP study that investigated the manifestation of diabetes in an adult Ugandan population with new-onset diabetes were randomly selected to join this study and then followed up for 12 months.

### Eligibility criteria: inclusion and exclusion criteria

The participants recruited in the study were those aged ≥ 18 years with a recent diagnosis of diabetes (< 3 months since diagnosis), clinically stable (without evidence of metabolic decompensation necessitating hospital admission), and had been initiated on OHA. The diagnosis of diabetes in all of these participants was made by clinicians at the various outpatient clinics based on the World Health Organisation (WHO) guidelines for the diagnosis of diabetes [10]. Pregnant women were excluded from this study.

### Assessment of demographic, clinical, biophysical, and metabolic characteristics at baseline and during the follow-up period

At baseline, information on relevant demographic (age at diagnosis and gender) and clinical characteristics (type of glucose-lowering treatment initiated at the time of diagnosis) were collected using a pre-tested case report form. This was followed by anthropometric (weight, height, waist circumference or WC, hip circumference, waist: hip circumference ratio or WHR, BMI) and resting blood pressure measurements.

A fasting blood sample was then collected for measurement of blood glucose (FBG), HbA1c, insulin, C-peptide, and lipid profile (total cholesterol or TC, high-density lipoprotein cholesterol or HDLC, low-density lipoprotein cholesterol or LDLC, triglycerides or TGL, and total cholesterol: high-density lipoprotein cholesterol ratio or TC/HDLC ratio). Insulin resistance (Homeostatic model assessment 2 for insulin resistance or HOMA2-IR) and the pancreatic beta-cell function (HOMA2-%B) were calculated using the online homeostatic model assessment-2 (HOMA2) calculator by the Diabetes Trial Unit of the University of Oxford, Oxford UK [11].

The study participants were then followed up every three months for 12 months. To maintain the participants in the study, the trained study nurses made periodic phone calls and also sent out phone messages to the participants reminding them of their scheduled appointments. These study nurses worked at the respective study sites and oversaw patient registration and diabetes education at the outpatient diabetes clinics.

Management of diabetes was determined by the attending clinicians following the WHO treatment guidelines which recommend the use of metformin as the first-line glucose-lowering drug and other oral agents like sulfonylureas or incretin therapies as add-on therapies [12]. At each time point, information on the type and dose of the OHA used, history of any hypoglycaemic episode regardless of severity and severe hyperglycaemia requiring hospital admission, and treatment adjustment (dose increase or addition of a new drug) was obtained. In addition, all participants were subjected to anthropometry and measurement of HbA1c, as a measure of assessing glycaemic control and response to treatment. At the 12-month follow-up time point, a fasting blood sample was drawn from all participants for measurement of FBG, HbA1c, insulin, C-peptide, and lipid profile.

### Definition of study outcomes

Diabetes progression at the 12-month time point was defined as HbA1c ≥ 8% when on ≥ 2 OHA and/or treatment intensification (initiation of insulin therapy, and/or the dose of > 1 oral drug increased, and/or addition of a second oral agent) [2, 3].

### Statistical analysis

The categorical and continuous variables describing all the study participants at baseline and 12-month time points of follow-up were expressed as proportions and medians with inter-quartile range (IQR), respectively. The HbA1c of the participants at each time point was expressed as a mean ± standard deviation. The changes in HbA1c based on the OHA used at each time point were determined and summarised in the form of a table and graph.

The prevalence of diabetes progression was expressed as a frequency with 95% confidence intervals. Simple descriptive statistics were used in the comparison of the demographic, anthropometric, and metabolic characteristics of participants who progressed (progressors) and those who did not (non-progressors) at 12 months. To identify the specific predictors of progression of type 2 diabetes, variables that have been reported in the literature to be associated with type 2 diabetes progression like age, gender, relevant markers of adiposity, and an atherogenic lipid profile (WC and TC/HDLC ratio), and pancreatic beta-cell function (fasting C-peptide and HOMA2-%B) were added to the model and logistic regression was performed. A *p*-value < 0.05 and a 95% confidence interval above 1 were considered statistically significant. All analyses were done using STATA statistical software version 15 College Station, TX: StataCorp LLC.

### Ethical approval

The study received ethical approval from the research ethics committee of the Uganda Virus Research Institute (GC/127/18/05/650) and the Uganda National Council of Science and Technology (HS 2431). All participating study sites offered administrative approval before the initiation of the study. All study participants recruited into the study provided written informed consent. The study was conducted in accordance with the ethical principles outlined in the Declaration of Helsinki and the Good Clinical Practice guidelines of the International Conference on Harmonisation.

## Results

The demographic, clinical, anthropometric, and metabolic characteristics of all study participants at baseline are summarised in Table 1.

**Table 1:** Comparison of demographic, clinical, anthropometric, and metabolic characteristics of the participants at baseline

Characteristic	All study participants (*n* = 207)	Metformin only (*n* = 78, 37.7%)	Metformin and sulfonylurea (*n* = 129, 62.3%)
Age (years)	49 (40–57)	50 (43–57)	49 (39–57)
Gender			
1. Male	101 (48.8)	37 (47.4)	55 (42.6)
2. Female	106 (51.2)	41 (52.6)	74 (57.4)
BMI (kg/m^2^)	27.5 (24.3–31.6)	27.2 (24.2–31.9)	27.7 (24.1–31.3)
WC (cm)	96.5 (89.0–104.0)	96.8 (88–106.4)	96.5 (89.0–104.0)
WHR	0.93 (0.89–0.96)	0.92 (0.89–0.97)	0.94 (0.90–0.96)
Systolic BP (mmHg)	127 (117–137)	128 (121–140)	125 (115–134)
Diastolic BP (mmHg)	84 (77–91)	87 (81–93)	82 (75–89)
HbA1c (%)	10.7 (8.3–12.3)	10.2 (8.3–12.3)	10.9 (8.4–12.5)
HbA1c (mmol/mol)	93 (67–111)	88 (67–111)	95 (68–113)
FBG (mmol/l)	8.6 (6.5–12.8)	8.8 (6.9–13.4)	8.2 (6.1–11.8)
Fasting insulin (µmol/l)	5.6 (3.2–10.0)	6.0 (3.6–10.0)	5.2 (3.0–9.6)
Fasting C-peptide (ng/ml)	1.4 (0.9–2.0)	1.4 (0.8–1.9)	1.3 (0.9–2.0)
Total cholesterol (mmol/l)	4.0 (3.2–5.0)	4.2 (3.2–5.3)	3.9 (3.2–4.9)
LDLC (mmol/l)	2.5 (1.9–3.5)	2.6 (2.0–3.6)	2.6 (1.9–3.5)
HDLC (mmol/l)	0.9 (0.7–1.2)	1.0 (0.8–1.2)	0.8 (0.7–1.1)
TGL (mmol/l)	1.5 (1.0–1.9)	1.5 (1.1–2.0)	1.4 (1.0–1.9)
Non-HDLC (mmol/l)	3.0 (2.3–4.0)	3.2 (2.2–4.3)	3.0 (2.4–3.8)
TC/HDLC	4.6 (3.6–5.5)	4.4 (3.6–5.1)	4.6 (3.7–5.6)
HOMA2-IR	1.1 (0.7–1.8)	1.1 (0.7–2.1)	1.1 (0.7–1.7)
HOMA2-%B	43.3 (21.6–69.2)	40.1 (15.7–57.9)	44.5 (24.6–70.5)

*BMI* Body mass index, *BP* Blood pressure, *FBG* Fasting blood glucose, *HbA1c* Glycated haemoglobin, *HDLC* High-density lipoprotein cholesterol, *HOMA2-IR* Homeostatic model assessment insulin resistance, *HOMA2-%B* Homeostatic model assessment pancreatic beta-cell function, *LDLC* low-density lipoprotein cholesterol, *TC* total cholesterol, *TGL* triglycerides, *TC/HDLC* total cholesterol: high-density lipoprotein cholesterol ratio, *WC* waist circumference, *WHR* waist: hip circumference ratio

At baseline, the median (IQR) age at diagnosis, BMI, and HbA1c for the participants were 49 years (40–57), 27.5 kg/m^2^ (24.3–31.6), and 93 mmol/mol (67–111) or 10.7% (8.3–12.3), respectively. Seventy-eight (37.7%) participants were on metformin monotherapy while 129 (62.3%) were on metformin and sulfonylurea combination. No participant was on 3 oral hypoglycaemic agents or insulin at baseline.

One hundred sixteen participants (56%) completed the follow-up at 12 months. Of these, 63 (54.3%) were female.

### Comparison of clinical, anthropometric, and metabolic characteristics of participants at baseline and 12 months

Compared with those at baseline, participants at the 12-month time point of follow-up had a higher median BMI (29 kg/m^2^ [25.1–31.7] vs 27.5 kg/m^2^ [24.3–31.6], *p* = 0.001). There was no statistically significant difference in most of the clinical and metabolic characteristics between both groups (Supplementary Table 1).

On stratification in two groups based on HbA1c cut-offs and number of OHA being used at baseline, compared with those with HbA1c ≥ 8% and on ≥ 2 OHA, participants with HbA1c < 8% and on < 2 OHA had a higher prevalence of pre-existing hypertension (51.2% vs 25%), a higher median (IQR) systolic blood pressure (139 [127–152] vs 125 [113–133] mmHg), and HOMA2-%B (70.8 [53.1–99.8] vs 38.7 [23.4–65.6]). No differences were noted in the anthropometric and most metabolic characteristics between both groups (Supplementary Table 2).

### Changes in HbA1c based on the number and type(s) of oral treatment used

**Fig. 1:**
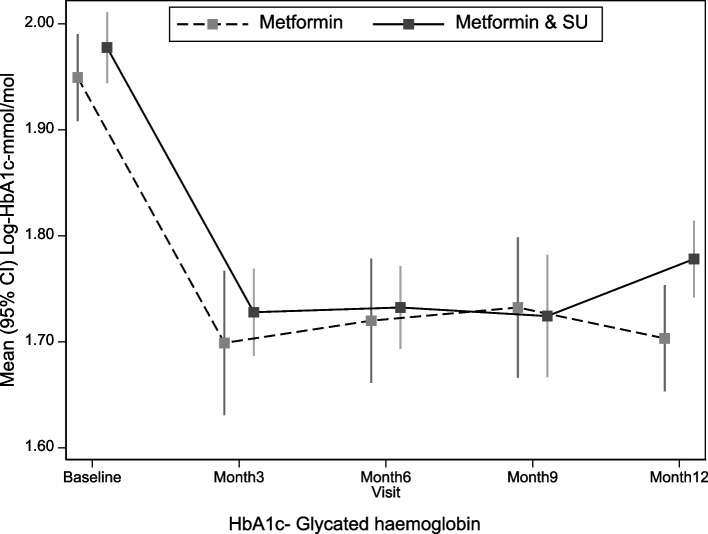
Mean (95% CI) log glycated haemoglobin changes over the follow-up time points based on the glucose-lowering treatment used. HbA1c- Glycated haemoglobin

The HbA1c changes of all study participants, based on the HbA1c cut-offs at baseline and OHA used at each time point are summarised in Supplementary Tables 3 and 4, and Fig. 1.

Both participants on metformin monotherapy and metformin-sulfonylurea combination experienced an initial sharp decline in HbA1c between the baseline and 3-month time points. However, this decline was maintained at the 6-and 9-month time points in participants on combination therapy, with a steady rise in HbA1c later between the 9- and 12-month time points (Fig. 1 and Supplementary Table 3).

Participants with HbA1c < 8% and on < 2 OHA at baseline had a steadily maintained median HbA1c level throughout the follow-up period while those who had HbA1c ≥ 8% and on ≥ 2 OHA experienced a gradual decline in the median HbA1c level (Supplementary Table 4).

### Diabetes progression after 12 months of follow-up

Tables 2 and 3 summarise the treatment adjustments at each time point and the independent predictors of short-term diabetes progression, respectively.

**Table 2:** Treatment adjustments at each time point

Time point	Treatment adjustment	Metformin only (n,%)	Metformin and sulfonylurea (n,%)
3 months (*n* = 131 participants)	-New drug added and/or the dose of > 1 drug increased	16, 35.6%	8, 10.7%
6 months (*n* = 92 participants)	-New drug added and/or the dose of > 1 drug increased	10, 40%	10, 16.7%
9 months (*n* = 67 participants)	-New drug added and/or the dose of > 1 drug increased	12, 50%	7, 21.2%
12 months (*n* = 116 participants)	-New drug added and/or the dose of > 1 drug increased	17, 39.5%	17, 27.9%

**Table 3:** Predictors of diabetes progression at 12 months of follow-up

Characteristic	Adjusted OR (95% CI)	*P*-value
Age at diagnosis (years)	0.9 (0.9–1.0)	0.13
Female gender	3.2 (1.1–9.2)	0.03
Waist circumference (cm)	1.1 (1.0–1.2)	0.08
TC/HDLC	1.1 (0.9–1.5)	0.30

*OR* Odds ratio, *TC/HDLC* Total cholesterol: high-density lipoprotein cholesterol

Of the 116 participants (56%) who completed the 12-month follow-up study period, diabetes progression was observed in 64 participants, corresponding to a prevalence of 55.2% (95% CI 45.7–64.4). An HbA1c ≥ 8% on ≥ 2 OHA and treatment intensification were noted in 44.8% and 29.3% of participants, respectively.

Of the 34 participants who had treatment intensification at the 12-month time point, 10 participants had a new drug added to their treatment regimen (sulfonylurea and pre-mixed insulin were added to the treatment regimen of nine participants and one participant, respectively).

Treatment adjustment at each time point of follow-up was documented more in participants initiated on metformin monotherapy at baseline (Table 2). Conversely, an HbA1c of ≥ 8% on ≥ 2 OHA was observed more in participants initiated on metformin and sulfonylurea combination at baseline compared with those initiated on metformin monotherapy (*n* = 34, 65.4% vs *n* = 18, 34.6% respectively).

On multivariate analysis, only the female gender (adjusted odds ratio or AOR 3.2, 95% CI 1.1–9.2, *p* = 0.03) was noted to independently predict diabetes progression at the 12-month time point of follow-up (Table 3).

### Comparison between diabetes progressors and non-progressors at 12 months of follow-up

Supplementary Table 5 summarises the demographic, clinical, anthropometric, and metabolic characteristics of participants who progressed and those who did not at the 12-month time point of follow-up.

Compared with the non-progressors, participants classified as progressors had a higher HbA1c level (69 mmol/mol [55–85] vs 47 mmol/mol [40–59]), HOMA2-IR (1.7 [1.2–2.5] vs 1.2 [0.9–2.0]), and a lower HOMA2-%B (39.8 [26.1–59.9] vs 90.3 [46.5–120.7]). No marked differences were noted in BMI, WHR, lipid profile, fasting insulin, and C-peptide concentrations between both groups.

On comparing the baseline characteristics of participants who completed the follow-up period and those who were lost to follow-up, the latter group had more females (62.5%) and had a lower median (IQR) HbA1c (9.3 [8.2–11.9] vs 11.4 [8.8–12.9] %, respectively) (Supplementary Table 6).

## Discussion

To our knowledge, this is the first study to investigate short-term progression of type 2 diabetes in an adult Ugandan population with new-onset diabetes. In this observational prospective study, we report that short-term diabetes progression occurred in sixty-four participants (55.2%, 95% CI 45.7–64.4) during a 12-month period of follow-up in an adult Ugandan population with newly diagnosed type 2 diabetes, and was independently associated only with the female gender. In addition, progressors had higher markers of glycaemia (FBG and HbA1c) and insulin resistance (HOMA2-IR) and reduced pancreatic beta-cell function (lower HOMA2-%B). These observations are in accord with observations from other populations [4–8].

Female gender has also been reported to be an independent predictor of diabetes progression in some studies [13–17] while others have reported that it may be protective [18, 19]. In one multi-ethnic prospective cohort study of 500 adult patients with type 2 diabetes, the female gender was associated with a two-fold increase in the subsequent use of insulin therapy during the three years of follow-up [13]. The reasons explaining the increased risk of diabetes progression in females are not well documented but may be related to having higher measures of body adiposity (increased WC, BMI) and an adverse lipid profile pattern (low HDLC with increased LDLC and TGL concentrations), which are associated with an increased risk of diabetes progression [8].

In contrast, we did not observe any association between most measures of adiposity like BMI and WC, adverse lipid profile patterns, and diabetes progression in our study population. Most studies performed in Caucasian populations have reported an association [4, 7, 13, 14, 19–29]. This might be due to methodological differences. For example, in some of these studies, diabetes progression was defined as early initiation of insulin therapy, glycaemic deterioration, and pancreatic beta-cell dysfunction and participants were followed up for a longer duration.

Prospective studies have demonstrated that diabetes progression is associated with both high [4, 13, 14] and low BMI [19, 22], reflecting an association with both extremes of BMI. A low BMI which reflects reduced pancreatic beta-cell secretory function is a common finding in adult African patients with type 2 diabetes and is often associated with the need for early initiation of insulin therapy [9, 30, 31]. This might also explain the lack of association between the risk of progression with other markers of the metabolic syndrome, such as those of lipid metabolism, in our study population.

### Strengths and limitations

To our knowledge, this is the first study to prospectively follow up a cohort of unselected adult African patients with new-onset diabetes to understand, in a real-world setting, the frequency and predictors of short-term progression of type 2 diabetes. Despite this strength, our study had some limitations. Its short follow-up period may explain why we did not observe an association between diabetes progression and commonly reported phenotypic characteristics like HbA1c, BMI, WC, and some lipid profile parameters. We also had a very high rate of loss to follow up through the study. This is because the study was mainly performed during the COVID-19 pandemic which significantly affected retention in clinical care due to the frequent and prolonged lockdown periods and challenges in accessing public transport for most participants. Additional factors associated with diabetes progression like lifestyle or environmental factors and specific genetic variants were not assessed in this study.

## Conclusion

In this study, we report that short-term diabetes progression, especially glycaemic deterioration, was relatively common in our study population and was associated only with the female gender. These findings underscore the need for early intensified individualised glycaemic management in female patients with newly diagnosed type 2 diabetes to avert rapid short-term diabetes progression.

### Supplementary Information

**Additional file 1: Supplementary Table 1.** Comparison of sociodemographic, clinical, anthropometric, and metabolic characteristics for the participants at baseline and 12 months of follow-up. **Supplementary Table 2.** Baseline characteristics of participants based on glycated haemoglobin cut-offs and number of oral hypoglycaemic agents used. **Supplementary Table 3.** Mean glycated haemoglobin changes for the participants over the follow-up period. **Supplementary Table 4.** Glycated haemoglobin changes during follow-up of the of participants based on glycated haemoglobin cut-offs and number of oral hypoglycaemic agents used. **Supplementary Table 5.** Sociodemographic, clinical, and metabolic characteristics of participants categorised according to the status of diabetes progression at 12 months. **Supplementary Table 6.** Baseline characteristics of participants who were lost to follow-up and those who completed the follow-up.

## Data Availability

The datasets used and/or analysed during the current study are available from the corresponding author upon reasonable request.

## Abbreviations

BMI: Body mass index
BP: Blood pressure
FBG: Fasting blood glucose
HbA1c: Glycated haemoglobin
HDLC: High-density lipoprotein cholesterol
HOMA2-IR: Homeostatic model assessment insulin resistance
HOMA2-%B: Homeostatic model assessment pancreatic beta-cell function
LDLC: Low-density lipoprotein cholesterol SSA-sub-Saharan Africa
TC: Total cholesterol
TGL: Triglycerides
TC/HDLC: Total cholesterol: high-density lipoprotein cholesterol ratio
UDIP: Uganda Diabetes Phenotype
WC: Waist circumference
WHR: Waist: hip circumference ratio
WHO: World Health Organisation
