# Supplementary material for: Type 2 diabetes progression in an adult Ugandan population with new-onset diabetes: an observational prospective study
Source: BMC Prim Care. 2023 Oct 19;24:214. doi: 10.1186/s12875-023-02169-4 (PMC10588137; doi:10.1186/s12875-023-02169-4)
Supplement: Supplementary file 1 — Additional file 1: Supplementary Table 1. Comparison of sociodemographic, clinical, anthropometric, and metabolic characteristics for the participants at baseline and 12 months of follow-up. Supplementary Table 2. Baseline characteristics of participants based on glycated haemoglobin cut-offs and number of oral hypoglycaemic agents used. Supplementary Table 3. Mean glycated haemoglobin changes for the participants over the follow-up period. Supplementary Table 4. Glycated haemoglobin changes during follow-up of the of participants based on glycated haemoglobin cut-offs and number of oral hypoglycaemic agents used. Supplementary Table 5. Sociodemographic, clinical, and metabolic characteristics of participants categorised according to the status of diabetes progression at 12 months. Supplementary Table 6. Baseline characteristics of participants who were lost to follow-up and those who completed the follow-up. [file 12875_2023_2169_MOESM1_ESM.docx]

**Supplementary Table 1. Comparison of sociodemographic, clinical, anthropometric, and metabolic characteristics for the participants at baseline and 12 months of follow-up**

| **Characteristic** | **Baseline (n=207)** | **At 12 months (n=116)** | **P value** |
| --- | --- | --- | --- |
| Age (years) | 49 (40-57) | 49 (40-56) | 1.00 |
| Sex 1. Male  2. Female | 101 (48.8)  106 (51.2) | 53 (45.7)  63 (54.3) | 0.40 |
| BMI | 27.5 (24.3-31.6) | 29 (25.1-31.7) | 0.001 |
| WC (cm) | 96.5 (89.0-104.0) | 96.5 (89-104) | 0.07 |
| WHR | 0.93 (0.89-0.96) | 0.94 (0.88-0.98) | 0.40 |
| Systolic BP (mmHg) | 127 (117-137) | 131 (123-140) | 0.17 |
| Diastolic BP (mmHg) | 84 (77-91) | 87 (81-92) | 0.21 |
| HbA1c (%) | 10.7 (8.3-12.3) | 7.6 (6.3-9.0) | 0.40 |
| HbA1c (mmol/mol) | 93 (67-111) | 59 (45-72) | 0.21 |
| FBG (mmol/l) | 8.6 (6.5-12.8) | 7.7 (5.6-10.8) | 0.47 |
| Fasting insulin (µmol/l) | 5.6 (3.2-10.0) | 10.8 (6.6-17.8) | 0.40 |
| Fasting C-peptide (ng/ml) | 1.4 (0.9-2.0) | 2.1 (1.6-2.9) | 0.45 |
| Total cholesterol (mmol/l) | 4.0 (3.2-5.0) | 4.7 (3.9-5.3) | 0.30 |
| LDLC (mmol/l) | 2.5 (1.9-3.5) | 2.8 (2.1-3.5) | 0.33 |
| HDLC (mmol/l) | 0.9 (0.7-1.2) | 1.0 (0.9-1.3) | 0.32 |
| TGL (mmol/l) | 1.5 (1.0-1.9) | 1.7 (1.5-2.4) | 0.64 |
| Non-HDLC (mmol/l) | 3.0 (2.3-4.0) | 3.6 (2.8-4.2) | 0.47 |
| TC/HDLC | 4.6 (3.6-5.5) | 4.5 (3.6-5.1) | 0.27 |
| HOMA2-IR | 1.1 (0.7-1.8) | 1.6 (1.1-2.3) | 0.31 |
| HOMA2-%B | 43.3 (21.6-69.2) | 46.5 (31.5-90.3) | 0.35 |

All categorical and continuous variables are reported as proportions and medians (IQR), respectively.

BMI-body mass index, BP-blood pressure, FBG-fasting blood glucose, HbA1c-glycated haemoglobin, HDLC- high-density lipoprotein cholesterol, HOMA2-IR: Homeostatic model assessment insulin resistance, HOMA2-%B- Homeostatic model assessment pancreatic beta-cell function, LDLC- low-density lipoprotein cholesterol, TC-total cholesterol, TGL- triglycerides, TC/HDLC- total cholesterol: high-density lipoprotein cholesterol ratio, WC-waist circumference, WHR-waist: hip circumference ratio

**Supplementary Table 2. Baseline characteristics of participants based on glycated haemoglobin cut-offs and number of oral hypoglycaemic agents used**

| **Characteristic** | **Participants with HbA1c <8% and on <2 OHA (n=41)** | **Participants with HbA1c ≥8% and on ≥2 OHA (n=64)** |
| --- | --- | --- |
| Age, years | 47 (39-50) | 48 (39-56) |
| Female sex, n (%) | 24 (58.5) | 35 (54.7) |
| Urban residence, n (%) | 32 (78.1) | 45 (70.3) |
| Pre-existing hypertension, n (%) | 21 (51.2) | 16 (25) |
| Systolic BP, mmHg | 139 (127-152) | 125 (113-133) |
| Diastolic BP, mmHg | 90 (85-95) | 82 (75-89) |
| BMI, kg/m^2^ | 27.0 (22.3-32.7) | 27.7 (25.1-31.3) |
| WC, cm | 95.3 (85.1-105.2) | 96.8 (89.0-104.0) |
| WHR | 0.90 (0.86-0.97) | 0.94 (0.90-0.96) |
| HbA1c, % | 6.3 (5.4-6.8) | 11.7 (10.0-13.2) |
| HbA1c, mmol/mol | 45 (35-51) | 104 (86-120) |
| Fasting blood glucose, mmol/l | 5.7 (5.1-7.3) | 9.1 (6.5-12.7) |
| Fasting insulin, µmol/l | 6.1 (4.4-9.6) | 5.0 (3.0-9.6) |
| Fasting C-peptide, ng/ml | 1.50 (0.93-2.08) | 1.30 (0.90-1.87) |
| TC, mmol/l | 4.0 (3.2-5.5) | 3.9 (3.2-4.9) |
| HDLC, mmol/l | 0.96 (0.85-1.16) | 0.85 (0.71-1.16) |
| TGL, mmol/l | 1.5 (0.8-2.3) | 1.3 (1.0-1.8) |
| LDLC, mmol/l | 2.4 (2.1-3.5) | 2.7 (1.9-3.5) |
| Non-HDLC, mmol/l | 3.1 (2.3-4.6) | 3.0 (2.4-3.9) |
| TC/HDLC | 4.1 (3.0-6.5) | 4.6 (3.7-5.6) |
| HOMA2-IR | 0.95 (0.71-1.56) | 1.16 (0.70-1.74) |
| HOMA2-%B | 70.8 (53.1-99.8) | 38.7 (23.4-65.6) |

All categorical and continuous variables are reported as proportions and medians (IQR), respectively.

BMI-body mass index, BP-blood pressure, FBG-fasting blood glucose, HbA1c-glycated haemoglobin, HDLC- high-density lipoprotein cholesterol, HOMA2-IR: Homeostatic model assessment insulin resistance, HOMA2-%B- Homeostatic model assessment pancreatic beta-cell function, LDLC- low-density lipoprotein cholesterol, TC-total cholesterol, TGL- triglycerides, TC/HDLC- total cholesterol: high-density lipoprotein cholesterol ratio, WC-waist circumference, WHR-waist: hip circumference ratio

**Supplementary Table 3. Mean glycated haemoglobin changes for the participants over the follow-up period**

|  | **Mean HbA1c changes at each time point** | | | | |
| --- | --- | --- | --- | --- | --- |
| **Diabetes therapies** | **Baseline**  **(mmol/mol±SD)** | **3 months (mmol/mol±SD)** | **6 months (mmol/mol±SD)** | **9 months**  **(mmol/mol±SD)** | **12 months**  **(mmol/mol±SD)** |
| Metformin and Sulfonylurea | 92±32 | 63±28 | 58±21 | 62±23 | 63±19 |
| Metformin only | 87±32 | 56±25 | 59±21 | 65±23 | 57±21 |
| All participants | 90±32 | 60±27 | 58±20 | 60±22 | 60±20 |

HbA1c- Glycated haemoglobin, SD- Standard deviation

**Supplementary Table 4. Glycated haemoglobin changes during follow-up of the of participants based on glycated haemoglobin cut-offs and number of oral hypoglycaemic agents used**

| **Time point of follow-up** | **Participants with HbA1c <8% and on <2 OHA (n=41)** | **Participants with HbA1c ≥8% and on ≥2 OHA (n=64)** |
| --- | --- | --- |
| Median (SD) HbA1c at baseline | 6.3 (5.4-6.8) | 11.7 (10.0-13.2) |
| Median (IQR) HbA1c at the 3-month time point | 6.4 (5.5-6.8) | 10.1 (8.6-12.4) |
| Median (IQR) HbA1c at the 6-month time point | 6.6 (6.2-7.1) | 9.5 (8.9-10.8) |
| Median (IQR) HbA1c at the 9-month time point | 6.9 (6.7-7.1) | 9.4 (9.0-9.9) |
| Median (IQR) HbA1c at the 12-month time point | 6.0 (6.5-6.8) | 9.6 (8.6-10.5) |

HbA1c- Glycated haemoglobin, IQR- Interquartile range, OHA- Oral hypoglycaemic agent, SD- Standard deviation

**Supplementary Table 5. Sociodemographic, clinical, and metabolic characteristics of participants categorised according to the status of diabetes progression at 12 months**

| **Characteristics** | **Progressors (n=64)** | **Non-progressors (n=52)** |
| --- | --- | --- |
| Age at diagnosis (years) | 47 (38-54) | 50 (43-58) |
| Sex Male  Female | 27 (42.2)  37 (57.8) | 26 (50)  26 (50) |
| Systolic BP (mmHg) | 128 (122-137) | 134 (128-144) |
| Diastolic BP (mmHg) | 87 (80-91) | 89 (82-92) |
| WC (cm) | 97.8 (90.2-104.0) | 95.0 (88.2-103.5) |
| WHR | 1.0 (0.9-1.0) | 0.9 (0.9-1.0) |
| BMI (kg/m^2^) | 28.4 (25.1-32.6) | 29.2 (25.0-31.2) |
| HbA1c (%) | 8.6 (7.3-10.1) | 6.5 (5.8-7.5) |
| HbA1c (mmol/mol) | 69 (55-85) | 47 (40-59) |
| FBG (mmol/l) | 9.1 (6.8-13.1) | 5.8 (4.8-7.6) |
| Fasting insulin (µmol/l) | 10.9 (7.3-16.7) | 9.1 (6.6-18.0) |
| Fasting C-peptide (ng/ml) | 2.2 (1.7-3.0) | 1.9 (1.5-2.7) |
| TC (mmol/l) | 4.7 (3.9-5.2) | 4.6 (3.9-5.3) |
| LDLC (mmol/l) | 3.0 (2.2-3.6) | 2.7 (2.0-3.5) |
| HDLC (mmol/l) | 1.0 (0.8-1.2) | 1.0 (0.9-1.3) |
| TGL (mmol/l) | 1.6 (1.5-2.1) | 1.9 (1.3-2.6) |
| Non-HDLC (mmol/l) | 3.7 (2.7-4.2) | 3.3 (2.8-4.2) |
| TC/HDLC | 4.5 (3.9-5.0) | 4.5 (3.2-5.4) |
| HOMA2-IR | 1.7 (1.2-2.5) | 1.2 (0.9-2.0) |
| HOMA2-%B | 39.8 (26.1-59.9) | 90.3 (46.5-120.7) |

All categorical and continuous variables are reported as proportions and medians (IQR), respectively.

BMI-body mass index, BP-blood pressure, FBG-fasting blood glucose, HbA1c-glycated haemoglobin, HDLC- high-density lipoprotein cholesterol, HOMA2-IR: Homeostatic model assessment insulin resistance, HOMA2-%B- Homeostatic model assessment pancreatic beta-cell function, LDLC- low-density lipoprotein cholesterol, TC-total cholesterol, TGL- triglycerides, TC/HDLC- total cholesterol: high-density lipoprotein cholesterol ratio, WC-waist circumference, WHR-waist: hip circumference ratio

**Supplementary Table 6: Baseline characteristics of participants who were lost to follow-up and those who completed the follow-up**

| **Characteristic** | **Participants lost to follow-up**  **(n=91)** | **Participants at the 12-month time point (n=116)** |
| --- | --- | --- |
| Age, years | 48 (40-56) | 50 (40-59) |
| Female sex, n (%) | 57 (62.6) | 63 (54.3) |
| Urban residence, n (%) | 62 (68.1) | 82 (70.7) |
| Pre-existing hypertension, n (%) | 33 (36.3) | 37 (31.9) |
| Systolic BP, mmHg | 129 (120-140) | 125 (115-134) |
| Diastolic BP, mmHg | 85 (79-92) | 83 (75-90) |
| BMI, kg/m^2^ | 27.9 (25.1-31.7) | 27.0 (23.5-31.2) |
| WC, cm | 99 (92-105) | 94 (87-104) |
| WHR | 0.94 (0.90-0.96) | 0.93 (0.89-0.96) |
| HbA1c, % | 9.3 (8.2-11.9) | 11.4 (8.8-12.9) |
| HbA1c, mmol/mol | 79 (66-107) | 101 (72-117) |
| FBG, mmol/l | 8.8 (6.3-11.2) | 8.5 (6.7-14.2) |
| Fasting insulin, µmol/l | 5.6 (3.5-10.5) | 5.2 (3.0-9.6) |
| Fasting C-peptide, ng/ml | 1.31 (0.94-1.97) | 1.55 (0.88-2.00) |
| TC, mmol/l | 3.8 (3.1-4.9) | 4.1 (3.2-5.0) |
| HDLC, mmol/l | 0.81 (0.69-1.01) | 0.98 (0.73-1.21) |
| TGL, mmol/l | 1.5 (1.1-2.0) | 1.4 (1.0-1.9) |
| LDLC, mmol/l | 2.4 (1.9-3.3) | 2.6 (2.1-3.8) |
| Non-HDLC, mmol/l | 8.9 (2.3-3.8) | 3.0 (2.4-4.1) |
| TC/HDLC | 4.6 (3.5-5.7) | 4.5 (3.6-5.1) |
| HOMA2-IR | 1.14 (0.71-1.89) | 1.05 (0.68-1.70) |
| HOMA2-%B | 43.1 (24.6-65.8) | 43.3 (16.3-70.3) |

All categorical and continuous variables are reported as proportions and medians (IQR), respectively.

BMI-body mass index, BP-blood pressure, FBG-fasting blood glucose, HbA1c-glycated haemoglobin, HDLC- high-density lipoprotein cholesterol, HOMA2-IR: Homeostatic model assessment insulin resistance, HOMA2-%B- Homeostatic model assessment pancreatic beta-cell function, LDLC- low-density lipoprotein cholesterol, TC-total cholesterol, TGL- triglycerides, TC/HDLC- total cholesterol: high-density lipoprotein cholesterol ratio, WC-waist circumference, WHR-waist: hip circumference ratio
